# Effect of intra-vaginal electric stimulation on bladder compliance in stress urinary incontinence patients: the involvement of autonomic tone

**DOI:** 10.3389/fnins.2024.1432616

**Published:** 2024-08-07

**Authors:** Hui-Hsuan Lau, Cheng-Yuan Lai, Ming-Chun Hsieh, Hsien-Yu Peng, Dylan Chou, Tsung-Hsien Su, Jie-Jen Lee, Tzer-Bin Lin

**Affiliations:** ^1^Division of Urogynecology, Department of Obstetrics and Gynecology, Mackay Memorial Hospital, Taipei, Taiwan; ^2^Department of Medicine, Mackay Medical College, New Taipei, Taiwan; ^3^Institute of Biomedical Sciences, Mackay Medical College, New Taipei, Taiwan; ^4^Department of Surgery, Mackay Memorial Hospital, Taipei, Taiwan; ^5^Institute of Translational Medicine and New Drug Development, College of Medicine, China Medical University, Taichung, Taiwan; ^6^Department of Physiology, School of Medicine, College of Medicine, Taipei Medical University, Taipei, Taiwan; ^7^Graduate Institute of Biomedical Electronics and Bioinformatics, National Taiwan University, Taipei, Taiwan

**Keywords:** viscoelasticity, thermodynamics, pudendal nerve, hypogastric nerve, intra-vaginal stimulation

## Abstract

**Objective:**

In addition to the well-established advantage that strengthened pelvic musculature increases urethral resistance in stress urinary incontinence (SUI) patients, intra-vaginal electrical stimulation (iVES) has been shown in preclinical studies to improve bladder capacity via the pudendal-hypogastric mechanism. This study investigated whether iVES also benefits bladder storage in SUI patients by focusing on compliance, a viscoelastic parameter critically defining the bladder’s storage function, in a clinical study. Moreover, the potential involvement of stimulation-induced neuromodulation in iVES-modified compliance was investigated by comparing the therapeutic outcomes of SUI patients treated with iVES to those who underwent a trans-obturator tape (TOT) implantation surgery, where a mid-urethral sling was implanted without electric stimulation.

**Patients and methods:**

Urodynamic and viscoelastic data were collected from 21 SUI patients treated with a regimen combining iVES and biofeedback-assisted pelvic floor muscle training (iVES-bPFMT; 20-min iVES and 20-min bPFMT sessions, twice per week, for 3 months). This regimen complied with ethical standards. Data from 21 SUI patients who received TOT implantation were retrospectively analyzed. Mean compliance (Cm), infused volume (Vinf), and threshold pressure (Pthr) from the pressure-flow/volume investigations were assessed.

**Results:**

Compared with the pretreatment control, iVES-bPFMT consistently and significantly increased Cm (18/21; 85%, *p* = 0.017, *N* = 21) and Vinf (16/21; 76%, *p* = 0.046; *N* = 21) but decreased Pthr (16/21; 76%, *p* = 0.026, *N* = 21). In contrast, TOT implantation did not result in consistent or significant changes in Cm, Vinf, or Pthr (*p* = 0.744, *p* = 0.295, *p* = 0.651, respectively; all *N* = 21).

**Conclusion:**

Our results provide viscoelastic and thermodynamic evidence supporting an additional benefit of iVES-bPFMT to bladder storage in SUI patients by modifying bladder compliance, possibly due to the potentiated hypogastric tone, which did not occur in TOT-treated SUI patients.

**Clinical trial registration:** ClinicalTrials.gov, NCT02185235 and NCT05977231.

## Highlights

- **Question:** It is unclear whether iVES-bPFMT provides therapeutic benefits for bladder storage in SUI patients and if this therapeutic effect is mediated by iVES-induced neuromodulation.
- **Findings:** It is iVES-bPFMT, not surgical sling repair, that has increased bladder compliance.
- **Meaning:** In addition to the well-recognized benefit to urethral resistance, iVES-bPFMT also modifies the viscoelasticity of the bladder itself, possibly by potentiating sympathetic hypogastric tone.

## 1 Introduction

The urinary bladder collects and stores urine before its disposal. As a highly compliant hollow organ, the bladder’s pressure is slightly elevated in response to considerable filling volume during urine storage (Sugaya et al., 2000). Compliance, which is calculated by the change in bladder volume divided by the change in pressure, is an important viscoelastic property that critically defines the storage function of the bladder (Bellucci et al., 2017). Impaired bladder compliance is deleterious because abnormally elevated intra-vesical pressure during bladder filling could lead to upper urinary tract damage (Wu and Franco, 2017) and/or a decrease in bladder capacity (Arunachalam and Heit, 2020).

Stress urinary incontinence (SUI) occurs when urine leaks involuntarily during exercise, coughing, sneezing, or physical exertion, due to weakness in the muscles and/or tissues surrounding the urethra (Abrams et al., 2003). In women over 70 years of age, SUI is a common problem affecting the physical, social, and hygienic aspects of their daily lives (Abrams et al., 2002). Since SUI is well recognized as being caused by impaired urethral resistance (Abrams et al., 2003), electric pelvic stimulation—a protocol that efficiently strengthens the musculature (Porcari et al., 2002)—is accepted as a non-invasive option for treating SUI (MacLachlan and Rovner, 2015; Rodrigues et al., 2019). Particularly, intra-vaginal electric stimulation (iVES) directly stimulates the pelvic floor muscles using an intra-vaginal electrode (Stania et al., 2022), and it is prescribed as a home-based regimen because modern portable devices make it feasible for patients to perform iVES programs at home (Ugurlucan et al., 2014). A Cochrane review has summarized that iVES effectively relieves symptoms/signs and improves the quality of life for SUI patients (Todhunter-Brown et al., 2022).

Notably, a clinical trial has demonstrated that iVES stimulates the pudendal nerve in patients while strengthening pelvic musculature (Rodrigues et al., 2019). Animal studies have revealed that electric stimulation of the external sphincter not only directly strengthens pelvic musculature but also increases voiding efficacy by activating the sensory pudendal nerve (Langdale and Grill, 2016). Moreover, a preclinical investigation has demonstrated that iVES potentiates sympathetic hypogastric tone (Lindström et al., 1983), and studies exploring the neural control of bladder capacity have shown that sensory pudendal nerve stimulation benefits bladder storage by increasing hypogastric nerve activity (Gonzalez and Grill, 2019). These several observations raise the hypothesis that, in addition to the well-characterized advantage in urethral resistance, iVES also benefits the bladder capacity of SUI patients through an underlying neural mechanism.

Therefore, in the current study, we first investigated whether iVES benefits bladder functions by focusing on compliance, a viscoelastic parameter characterizing the bladder’s storage function. Additionally, since it is challenging to invasively record nerve activity in clinical settings, researchers investigated the potential involvement of neural modulation in iVES-modified bladder function by comparing bladder compliance in response to iVES therapy and trans-obturator tape (TOT) implantation (Medina et al., 2017). As a mid-urethral sling, TOT increases urethral resistance (De Leval, 2003) without applying electric current to the pelvic cavity of SUI patients.

Moreover, to comply with the principle of beneficence in ethics, we prescribed a regimen combining iVES with biofeedback-assisted pelvic floor muscle training (bPFMT; iVES-bPFMT) to obtain the maximal therapeutic effect. Among the various regular and modified pelvic floor muscle training methods, bPFMT is recognized as a home-based option that benefits the self-management of SUI patients (Perrier and Aumont, 2024) and has been shown to produce satisfactory therapeutic outcomes (Wu et al., 2021).

## 2 Methods

### 2.1 Patients

This study complied with the Declaration of Helsinki. The protocols were reviewed, approved, and periodically monitored by the ethics committee of Mackay Memorial Hospital, Taipei, Taiwan (23MMHIS058e and 20MMHIS410e) and were registered on ClinicalTrials.gov (NCT05977231 and NCT05255289). Pressure-flow studies of 45 female SUI patients (<75 years old; 23 patients received iVES-bPFMT and 22 patients received TOT implantation) were retrospectively reviewed. Patients diagnosed with pelvic organ prolapse or showing storage symptoms other than SUI were excluded from the study. No patient reported symptom(s)/sign(s) affecting the result of urodynamic investigations. Two patients received iVES-bPFMT but their pressure-volume analysis failed to form into a complete loop, and one patient received TOT whose compliance of bladder filling cannot be defined was excluded. Thus, a total of 21 iVES-bPFMT-treated and 21 TOT-treated patients were included in the statistical analysis.

### 2.2 Intra-vaginal electric stimulation

Intra-vaginal electric stimulation (iVES) was performed using a stimulating device (FemiScan; Mega Electronics, Kuopio, Finland) with a vaginal electrode probe. The stimulation parameters were as follows: frequency, 35 Hz; pulse width, 250 μS; duty time, 5 s; duty interval, 10 s; total stimulation time, 20 min; and current strength: just below the patient’s maximal tolerable intensity (maximum 100 mA).

### 2.3 Biofeedback-assisted pelvic floor muscle training

Biofeedback-assisted pelvic floor muscle training (bPFMT) was conducted using a wireless module (Medical Measurement System, Enschede, Netherlands) that allows the patient to hear instructions and feedback via a headphone during home practices. Each training session generally started at 1 min and increased in duration to 7 min, and it was repeated twice daily.

### 2.4 Trans-obturator tape

The procedure of trans-obturator tape (TOT) was conducted following Delorme’s method (Delorme, 2001). In brief, after the surgical area was sterilized and a Foley catheter was inserted in the lithotomy position, a small incision was made in the vagina, and two small cuts were made in the groin muscles on both sides. Using a needle, each end of the sling was guided from the vagina and passed through the obturator foramen to the groin muscles. The ends of the sling were cut off once it was confirmed to be in the correct position.

### 2.5 Cystometry investigation

Protocols for cystometry complied with the guidelines of the International Continence Society (ICS) (Schafer et al., 2002). Briefly, a multi-channel urodynamic study was conducted in which warm saline (37°C) was infused (80 mL/min) into the patients’ bladders. The data were recorded (MMS UD-200, Medical Measurement System, Enschede, Netherlands) and analyzed (Biopac MP36, Biopac Systems, Santa Barbra, US) using computer systems. The vesical pressure (Pves), abdominal pressure (Pabd), detrusor pressure (Pdet), urethral flow (Flow), voided volume (Vvod), infused volume (Vinf), and intra-vesical volume (Vive) were recorded online.

### 2.6 Pressure-volume analysis

**Figure 1:**
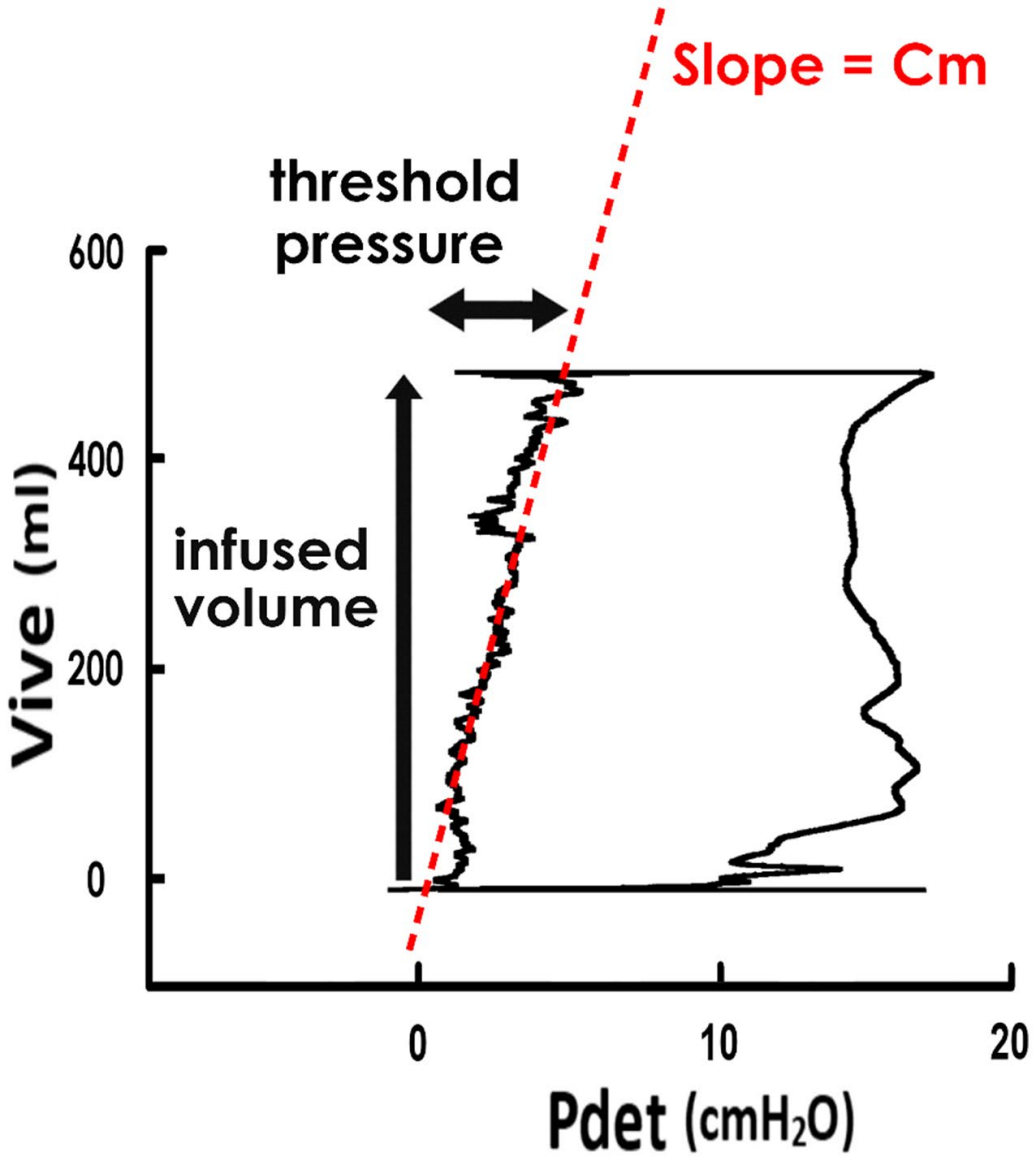
Pressure-volume analysis and derived parameters. Pressure-volume analysis of voiding was established by plotting the intra-vesical volume (Vive) versus the detrusor pressure (Pdet). The trajectory of data points forms a loop representing a voiding cycle, and the left boundary of the loop indicates the filling stage. The mean compliance of the filling stage (Cm) is calculated as the slope of the regression line of the left bolder (red dash line). The infused volume of the voiding cycle is represented by the level of the upper bolder, and the threshold pressure is done by the Pdet at the end-point of the filling stage.

Derived from the cystometry, the pressure-volume analysis (PVA) of voiding cycles was established by plotting Vive versus Pdet (Figure 1). The mean compliance (Cm) during the filling stage was calculated as the slope of the regression line of the filling stage (i.e., the left bolder of the trajectory loop). The level of the top bolder represented the infused volume (Vinf), and the Pdet at the end of the filling stage represented the threshold pressure.

### 2.7 Statistical analyses

The demographic and perioperative characteristics of patients were summarized using descriptive statistics. The measurement data were expressed as mean ± SEM. The difference in values between groups was assessed using paired Student’s t-tests. A significance in difference was set at a *p*-value of < 0.05.

## 3 Results

### 3.1 Patients’ database

A total of 42 female SUI patients were eligible for statistical analysis in this study. The mean age of the 21 patients who received iVES-bPFMT was 55.52 ± 3.06 years, and the mean age of 21 patients who received TOT implantation was 56.19 ± 2.82 years, which indicated that there was no statistical difference (*p* = 0.633, *N* = 21). Urodynamic evaluations were carried out, on average, 27.28 ± 5.62 days before starting iVES-bPFMT and 201.33 ± 43.96 days after completing iVES-bPFMT. Additionally, evaluations occurred 33.71 ± 8.75 days before and 191.05 ± 29.90 days after the TOT operation.

### 3.2 iVES-bPFMT increased Cm

**Figure 2:**
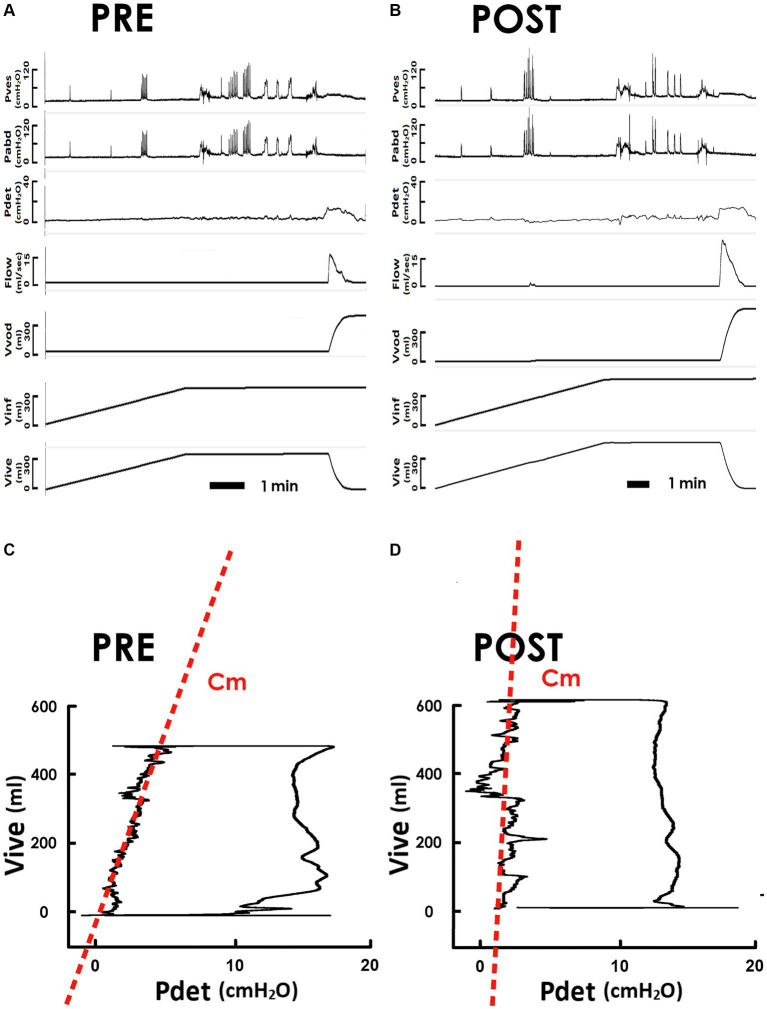
Pressure-flow and pressure-volume analyses of the voiding in response to iVES-bPFMT. **(A,B)** Representative cystometry of an SUI patient measured before and after iVES-bPFMT (PRE and POST, respectively). Pves: vesical pressure, Pabd: abdominal pressure, Pdet: detrusor pressure, Flow: urethral flow, Vvod: voided volume, Vinf: infused volume, and Vive: intra-vesical volume. **(C,D)** Pressure-volume analyses of pre- and post-treatment voiding cycles. The compliance of the filling stage (Cm) is measured as the slope of the regression lines (red dashed lines) of the left bolder of the loop.

First, to clarify the impact of iVES-bPFMT on bladder compliance, we compared pressure-volume analyses (PVAs) of SUI patients measured before and after the treatment. Derived from the pre- and post-treatment cystometry (Figure 2A, PRE and Figure 2B POST, respectively), PVAs were established by plotting the intra-vesical volume (Vive) versus the detrusor pressure (Pdet; Figure 2C, PRE and 2D POST). The trajectory of each PVA formed a loop that represents a voiding cycle. The left bolder of the loop, where Vive increased with a small amount of Pdet increment, characterized bladder filling. The slope of the regression lines on the left bolder (red dash lines) represents the mean compliance of bladder filling (Cm).

**Figure 3:**
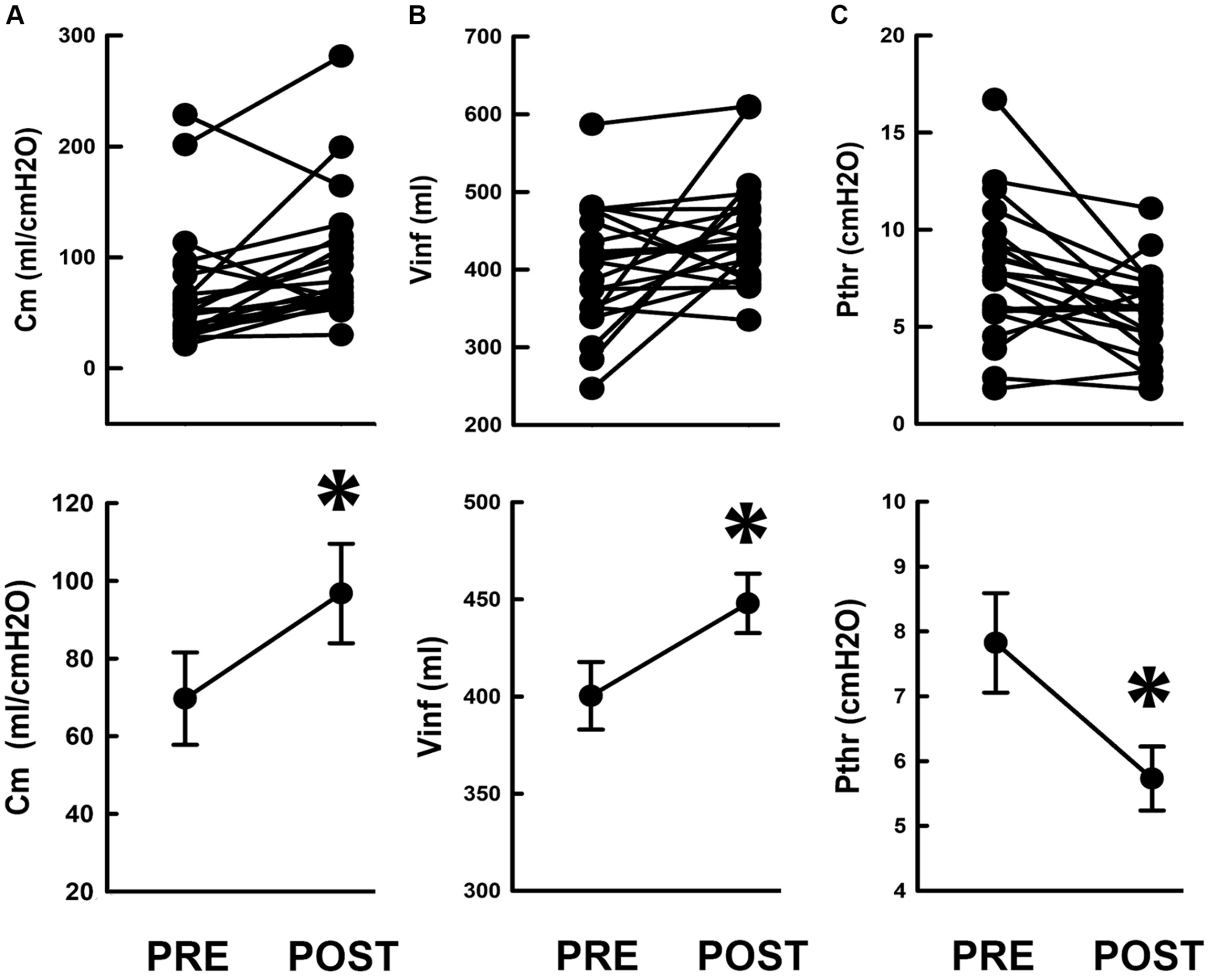
Compliance and associated parameters in response to iVES-bPFMT. **(A–C)** Individual (upper) and mean (lower) values of, respectively, the **(A)** compliance (Cm), **(B)** infused volume (Vinf), and **(C)** threshold pressure (Pthr) measured pre- and post-treatment (PRE and POST; *p = 0.017, *p* = 0.046, *p* = 0.026, respectively vs. PRE; all *N* = 21).

Compared to the pretreatment control, iVES-bPFMT markedly tilted the regression line anticlockwise, resulting in an elevated slope indicating an increased Cm after treatment. The Cm increment was confirmed by statistical analyses, which demonstrated that iVES-bPFMT consistently increased Cm in most patients (Figure 3A, upper, 18/21; 85%) and significantly increased the mean Cm of the patient (Figure 3A, lower, *p* = 0.017 vs. PRE; *N* = 21) compared to the pretreatment control.

### 3.3 iVES-bPFMT increased Vinf and decreased Pthr

Having observed that iVES-bPFMT increased the Cm of SUI patients, we then investigated the impact of iVES-bPFMT on the infusion volume (Vinf) and threshold pressure (Pthr) because Cm is defined as Vinf divided by Pthr.

Compared with the pretreatment PVA (Figure 2C), the level of the upper bolder of the loop, which represents Vinf, was elevated after iVES-bPFMT (Figure 2D). The treatment-induced increase in Vinf was confirmed by the summarized data showing that iVES-bPFMT consistently increased Vinf in most patients (Figure 3B, upper, 16/21; 76%) and significantly increased the mean Vinf of the patient (Figure 3B, lower, *p* = 0.046 vs. PRE; *N* = 21).

Moreover, compared to the pretreatment control (Figure 2C), the Pdet value at the end of filling, which represents Pthr, decreased after iVES-bPFMT (Figure 2D). The treatment-induced decrease in Pthr was confirmed by the summarized data demonstrating that iVES-bPFMT consistently decreased Pthrs in most patients (Figure 3C, upper, 16/21; 76%) and significantly decreased the mean Pthr of the patient (Figure 3C, lower, *p* = 0.026 vs. PRE; *N* = 21). These results collectively reveal that the iVES-bPFMT-associated Cm increment resulted from an increase in Vinf accompanied by a decrease in Pthr.

### 3.4 Unaffected Cm in response to TOT implantation

**Figure 4:**
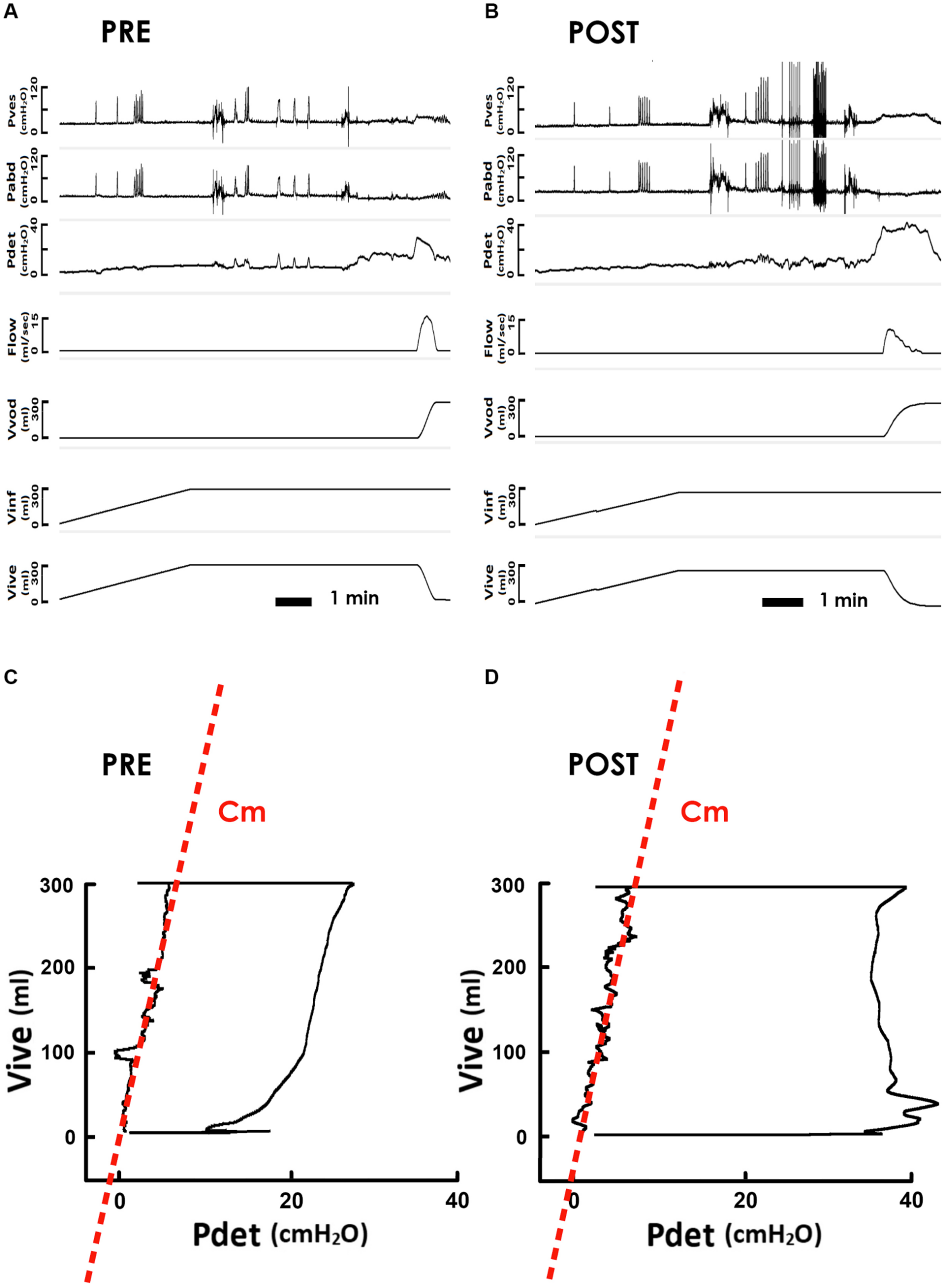
Pressure-flow and pressure-volume analyses of the voiding in response to TOT. **(A,B)** Representative cystometry of an SUI patient measured before and after the TOT repair (PRE and POST, respectively). Pves: vesical pressure, Pabd: abdominal pressure, Pdet: detrusor pressure, Flow: urethral flow, Vvod: voided volume, Vinf: infused volume, and Vive: intra-vesical volume. **(C,D)** Pre- and post-operative pressure-volume analyses of voiding cycles. The compliance of the filling stage (Cm) is measured as the slope of the regression lines (red dashed lines) of the left bolder of the loop.

To investigate the potential involvement of neural mechanisms in iVES-bPFMT, we investigated the impact of TOT operation on the bladder Cm of SUI patients because TOT has been shown to increase urethral resistance (Waltregny et al., 2008; Lau et al., 2022) without applying electric current to SUI patients. Derived from the pre- and post-operatively measured cystometry (Figure 4A, PRE and Figure 4B POST, respectively), representative PVAs demonstrated that, compared with the preoperative control (Figure 4C, PRE), TOT implantation barely affected the slope of the regression line of the filling stage (red dash line), implying that Cm remained relatively unaltered after the surgery (Figure 4D, POST).

**Figure 5:**
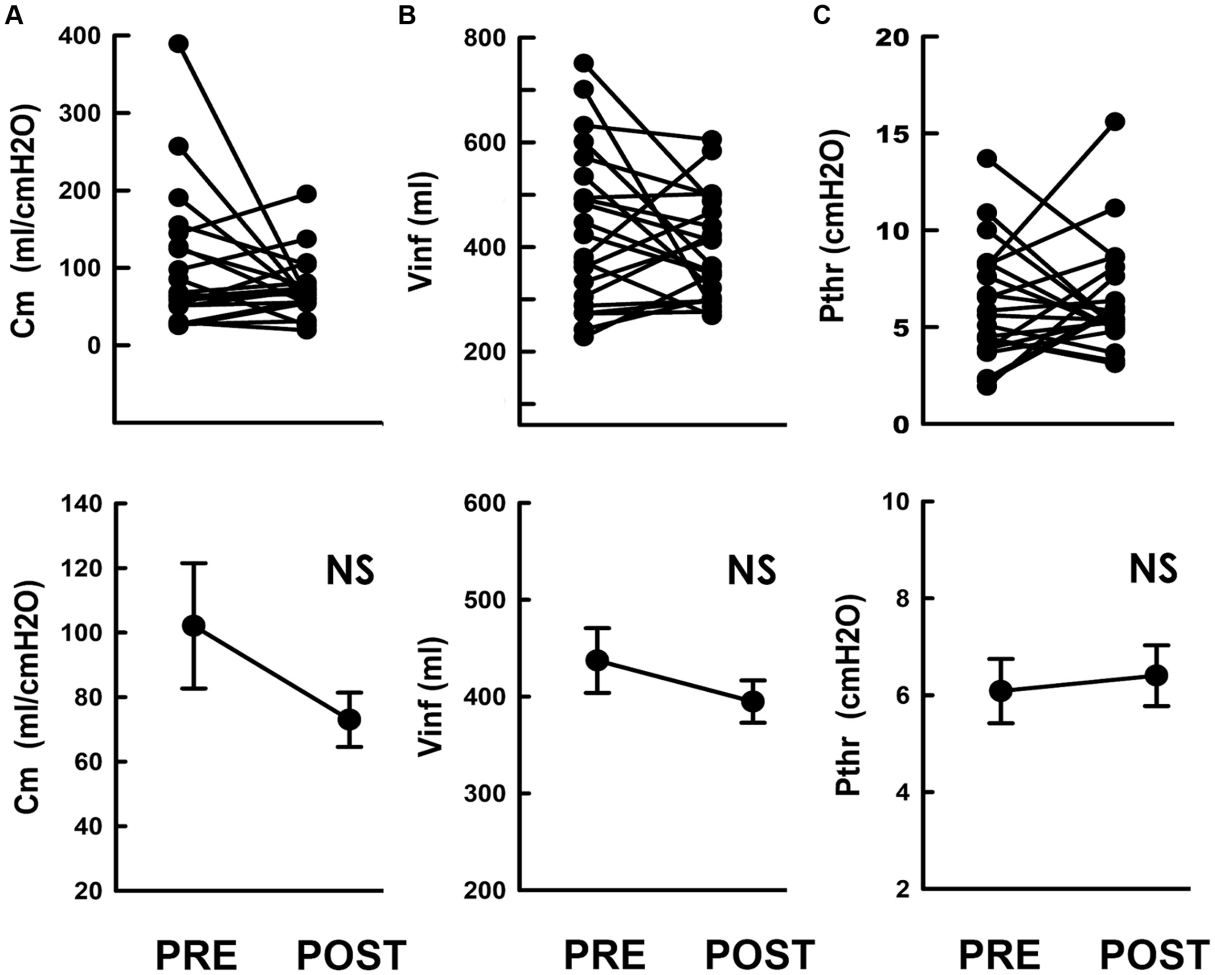
Compliance and associated parameters in response to TOT. **(A–C)** Individual (upper) and mean (lower) values of the **(A)** compliance (Cm), **(B)** infused volume (Vinf), and **(C)** threshold pressure (Pthr) measured pre- (PRE) and post-surgically (POST; NS *p* = 0.744, *p* = 0.295, *p* = 0.610, respectively vs. PRE; all *N* = 21).

The unaffected Cm in response to TOT implantation was confirmed by the statistical analysis, which demonstrated that this procedure neither consistently affected Cm in patients (Figure 5A, upper) nor significantly modified the mean Cm in patients (Figure 5A, lower, *p* = 0.744 vs. PRE; *N* = 21) compared to the preoperative control.

### 3.5 Unaffected Vinf and Pthr in response to TOT implantation

Subsequently, we investigated the impact of TOT procedure on Vinf and Pthr. Compared to the pre-operative PVA (Figure 4C), the level of the upper bolder of the loop, i.e., Vinf, remained relatively unchanged after TOT procedure (Figure 4D). Statistical analyses confirmed this result by demonstrating that TOT procedure failed to consistently alter Vinf in patients (Figure 5B, upper) or significantly affect the mean Vinf of patients (Figure 5B, lower, *p* = 0.295 vs. PRE; *N* = 21).

Moreover, when compared with the pre-operative control (Figure 4C), the Pdet at the endpoint of filling, i.e., Pthr, was not markedly altered by TOT surgery (Figure 4D). The unaffected Pthr after the operation was confirmed by statistical analyses showing that TOT neither consistently altered individual Pthr values of patients (Figure 5C, upper) nor significantly decreased the mean Pthr of patients (Figure 5C, lower, *p* = 0.651 vs. PRE; *N* = 21). These results reveal that unaffected Vinf and Pthr were associated with unmodified Cm after TOT procedure.

## 4 Discussion

### 4.1 iVES-bPFMT benefits bladder storage

In contrast to the well-established notion that the primary aim of pelvic floor stimulation for SUI patients is to strengthen the urethral sphincter muscle to increase urethral closure pressure (Sand et al., 1995), the current study investigated whether iVES also benefits bladder storage by modifying the viscoelasticity of the bladder itself via potential neural mechanisms.

Similar to questionnaire analyses showing improved quality of life for SUI patients with iVES (Stania et al., 2022) and bPFMT (Kulaksizoğlu et al., 2015; Wu et al., 2021), the current study provides viscoelastic and urodynamic evidence that iVES-bPFMT benefits bladder function by showing that this method increased compliance, a viscoelastic parameter critically characterizing the storage function of the bladder.

Compliance is defined as the change in infusion volume divided by the change in threshold pressure. Our results revealed that, accompanied by increased compliance, iVES-bPFMT increased the infusion volume and diminished threshold pressure. Since the infusion volume represents bladder capacity, an increase in this volume indicates that iVES-bPFMT improves bladder storage in SUI patients. Although the infusion volume increased after iVE-bPFMT, it did not result in an elevated threshold pressure; in fact, the threshold pressure decreased after treatment. Considering that an aberrantly elevated intravesical pressure in response to urine filling can lead to vesicoureteral reflux that results in upper urinary tract damage (Wu and Franco, 2017), our findings collectively suggest that iVES-bPFMT increases bladder compliance, which not only enhances bladder capacity to benefit continence but also avoids the potential risks of vesicoureteral reflux in SUI patients.

### 4.2 TOT surgery impacts negligibly on compliance

In the current study, PVA analyses reveal that **TOT surgery** has minimal impact on bladder compliance; this finding is supported by the result that both the infused volume and threshold pressure remained unmodified after TOT implantation. Building on the findings of a 3-year follow-up study (Waltregny et al., 2008) and our previous publication (Lau et al., 2022), which consistently demonstrated that TOT alleviates incontinence in SUI patients by effectively increasing outlet resistance during voiding, and similar clinical observations demonstrating that TOT procedure displays satisfactory relief of SUI-associated syndromes (Costantini et al., 2016), the findings of this study suggest that the effectiveness of the TOT procedure in improving continence in SUI patients is minimally attributed to its impact on the viscoelasticity of the bladder itself.

### 4.3 Discrepancy in effects on compliance

The mechanism underlying the discrepancy in the effects on bladder compliance between iVES-bPFMT and TOT procedure remains unclear. However, it is worth noting that, despite the therapeutic rationales of iVES-bPFMT (MacLachlan and Rovner, 2015) and TOT (Petros, 2022) methods aiming to improve continence in SUI patients by increasing urethral resistance during storage, thereby reducing episodes of urine incontinence (Rovner and Wein, 2004), the mechanisms of these treatments are not completely coincident. iVES-bPFMT trains/strengthens the musculature of the pelvic floor, allowing the trained/strengthened muscles to contract more efficiently during storage and relax well during voiding. In contrast, TOT surgically adds an exogenous physical barrier to the urethra that does not behave like the trained/strengthened muscle; it increases voiding resistance, benefiting storage, but it could impede urine emission because the surgically implanted sling does not relax during voiding. This point is supported by a clinical study demonstrating that the workload of voiding in SUI patients is increased after TOT implantation (Lau et al., 2022).

### 4.4 Potential neural mechanisms

Although the rationale behind how training/strengthening the pelvic floor muscles by iVES-bPFMT results in modified viscoelastic properties of the bladder itself is unclear, preclinical studies have revealed that electrical stimulation of the external sphincter muscle increases voiding efficacy via the activation of the sensory pudendal nerve (Langdale and Grill, 2016).

A clinical trial has demonstrated that iVES efficiently activates the pudendal nerve in patients (Rodrigues et al., 2019). Moreover, iVES has been shown to potentiate sympathetic hypogastric tone in cats (Lindström et al., 1983), and studies investigating the neural control of bladder capacity in rats have revealed that sensory pudendal nerve stimulation benefits bladder storage by enhancing hypogastric nerve activity (Gonzalez and Grill, 2019).

Given that preclinical studies have linked the pudendal-hypogastric mechanism to pelvic stimulation-modulated bladder capacity and that enhanced hypogastric nerve activity ultimately leads to detrusor relaxation, thereby decreasing intra-vesical pressure (Griffiths, 2004), we investigated the potential role of hypogastric tone in iVES-PFMT-increased compliance by assaying the detrusor pressure during filling. Our results reveal that, along with an increase in compliance, iVES-bPFMT decreased the threshold pressure, despite an increase in filling volume. Collectively, these results support our hypothesis that, in addition to strengthening the urethra musculature, iVES-bPFMT diminished bladder compliance, possibly via hypogastric tone-reduced bladder pressure.

### 4.5 TOT implantation failed to activate the neural response

Although the surgical implantation of TOT is recognized to sufficiently increase urethral resistance and thus improve continence in SUI patients (De Leval, 2003), the possibility that TOT implantation would activate potential neural mechanisms to modify bladder viscoelasticity is minimal because our data demonstrate that TOT procedure failed to affect bladder compliance, which was further confirmed by unmodified infusion volume and threshold pressure after TOT repair. Particularly, since an enhanced hypogastric tone would ultimately relax the detrusor and reduce the intra-vesical pressure (Griffiths, 2004), our results demonstrated that the threshold pressure remained unchanged after TOT implantation. Overall, these findings indicate that the discrepancy in bladder compliance between iVES-bPFMT and TOT methods could be attributed to the fact that iVES-bPFMT, and not TOT, activates the neural mechanism underlying the modification of bladder viscoelasticity. Therefore, the therapeutic benefit of iVES-bPFMT on bladder compliance likely involves, at least in part, the activation of the neural mechanism mediating the modification of bladder viscoelasticity.

As shown in Figures 3A, 5A, the preoperative compliance of almost all patients in both the iVES-bPFMT and TOT groups was above normal limits (20 mL/cmH2O). Thus, specifically improving bladder compliance may not be necessary for SUI therapy, as increasing urethral resistance is the primary goal (Sand et al., 1995).

Nevertheless, our findings in this study reveal that, in addition to the well-established advantage of increasing outlet resistance, iVES-bPFMT offers an additional benefit to continence by modifying bladder compliance via a potential neural mechanism not involved in the therapeutic outcome of TOT implantation. Our findings suggest the potential application of electric pelvic stimulation (with possible modifications) to the therapy of other storage/voiding conditions, such as neurogenic bladder and/or over-activated bladder, via neural mechanisms. Moreover, this approach is not restricted to urinary problems, as the hypogastric nerve is also involved in the physiology of defecation (Heitmann et al., 2021). If iVES can be used to treat bowel disorders, such as fecal urgency after a stapled hemorrhoidopexy (Calomino et al., 2011), it presents an interesting avenue that warrants further research.

### 4.6 Potential limitations

Although the involvement of iVES-potentiated hypogastric tone partly explains the neural mechanism underlying the effects observed in this study, other potential mechanisms, such as the inhibition of parasympathetic ganglionic (McGee et al., 2014), smooth muscle relaxation (Kadow et al., 2016), or modified central reflexes (Steers et al., 1988), have also been linked to pudendal nerve stimulation-inhibited bladder activity. Therefore, the possible involvement of these candidate mechanisms in iVES-diminished bladder compliance warrants further research.

In addition, to comply with the principle of beneficence, which is one of the four pillars of ethics, we have opted to prescribe iVES-bPFMT instead of iVES or bPFMT alone as a regimen to obtain a maximal therapeutic effect on the continence of SUI patients.

Nevertheless, because the possibility that the increase in compliance in this study could result from bPFMT alone or specifically from iVES-bPFMT cannot be completely excluded, future studies focusing on the effects of iVES or bPFMT alone are necessary to provide more information about the precise effect of conservative pelvic strengthening exercises on bladder compliance.

Finally, as a retrospective study using existing data, this study has inherent limitations in terms of internal and external validity. Moreover, since the sample size is relatively small, the potential bias in the effect of measurements cannot be neglected.

## 5 Conclusion

Our results provide viscoelastic and thermodynamic evidence supporting that, in addition to the well-recognized therapeutic advantage of strengthening the pelvic musculature, iVES-bPFMT also improves bladder storage in SUI patients by increasing bladder compliance. Moreover, by comparing the impacts of iVES-bPFMT and TOT procedure on compliance, we suggest that the iVES-bPFMT-modified bladder compliance is possibly attributed to the potentiated hypogastric tone, which does not occur in TOT-repaired SUI patients.

## Data availability statement

The raw data supporting the conclusions of this article will be made available by the authors, without undue reservation.

## Ethics statement

The studies involving humans were approved by the ethics committee of Mackay Memorial Hospital, Taipei, Taiwan (23MMHIS058e and 20MMHIS410e). The studies were conducted in accordance with the local legislation and institutional requirements. Written informed consent for participation was not required from the participants or the participants’ legal guardians/next of kin in accordance with the national legislation and institutional requirements.

## Author contributions

H-HL: Conceptualization, Writing – original draft, Writing – review & editing. C-YL: Data curation, Formal analysis, Investigation, Writing – original draft. M-CH: Data curation, Formal analysis, Investigation, Writing – original draft. H-YP: Data curation, Formal analysis, Investigation, Writing – original draft. DC: Data curation, Formal analysis, Methodology, Writing – original draft. T-HS: Writing – original draft. J-JL: Writing – original draft. T-BL: Writing – review & editing.
